# Case of Cerebral Inflammation Successfully Treated by Leeches

**Published:** 1819-08

**Authors:** E. Sutliffe


FOR THE LONDON MEDICAL AND PHYSICAL JOURNAL.

### *Case of Cerebral Inflammation successfully treated by Leeches.*

By E. SUTLIFFE, Esq.

THIS circumstance would not have been deemed worthy of notice, but for the peculiar stress laid upon topical applications to the base of the cranium and the nape of the neck, by Dr. Porter, of Bristol.

Master E. F. of Friday-street, æt. 4, had laboured under ardent fever for several days before I saw him. By the parents, his disease was thought to be simply dentition; and I believe I should not at that period have been called in, but another of the household was under my care. However, upon inspection, I found terrific symptoms, viz. a pulse full and throbbing, (the peculiar characteristic of cerebral inflammation,) face deeply and generally flushed, head excessively hot, great sensibility to noise, light, &c. My prognosis to the parents was unfavourable. I conceived it disputable whether the disease had not arrived at the incurable point of effusion, insomuch that I was hesitating on the use of means, anticipating a fatal issue; and, from the vigour of the depletory measures necessary to be resorted to, I feared lest we should be reflected upon as having hastened dissolution. This difficulty being got rid of, I advised several leeches to be applied to the temples, which bled for fourteen or sixteen hours, (this method succeeding in another case a few months prior to the present.) I added pulv. antimon. and calomel very largely, and great relief was quickly visible. On the third day after, the symptoms showed themselves with their original violence; but, a recurrence to the leeches again at the temples on one occasion, sufficed for every purpose, and convalescence rapidly supervened.

*Queen-street; May* 28, 1819.

